# Investigating the importance of recreational roads as a sediment source in a mountainous catchment using a fingerprinting procedure with different multivariate statistical techniques and a Bayesian un-mixing model

**DOI:** 10.1016/j.jhydrol.2018.12.019

**Published:** 2019-02

**Authors:** Kazem Nosrati, Adrian L. Collins

**Affiliations:** a Department of Physical Geography, School of Earth Sciences, Shahid Beheshti University, 1983969411 Tehran, Iran; b Sustainable Agriculture Sciences Department, Rothamsted Research, North Wyke, Okehampton EX20 2SB, UK

**Keywords:** Geochemical tracers, Modified MixSIR Bayesian model, Sediment source tracing, Statistical techniques

## Abstract

Road construction associated with land development generally increases erosion and sediment yields. Construction of unpaved roads has the potential to alter hydro-sedimentological behavior and catchment sediment source dynamics and, to date, this has largely been investigated in forested environments. The objective of this study, therefore, was to assess the relative importance of unpaved recreational roads as a sediment source alongside hillslope surface soils and stream channel banks in a non-forested mountainous catchment in northern Tehran, Iran, using a fingerprinting procedure. Eleven geochemical tracers were measured on 27 samples collected to characterise the sediment sources and five suspended sediment samples collected at the study catchment outlet. The statistical analysis employed to select three different composite fingerprints for discriminating the sediment sources comprised: (1) the Kruskal–Wallis H test (KW-H), (2) a combination of KW-H and discriminant function analysis (DFA), and (3) a combination of KW-H and principal components & classification analysis (PCCA). A Bayesian un-mixing model was used to ascribe sediment source contributions using the three composite fingerprints. Using the KW-H composite signature, the respective relative contributions (with uncertainty ranges) from recreational roads, hillslope surface soils and channel banks were estimated as 64.5% (57.7–73.1), 1.1% (0.1–4.9), and 33.9% (24.9–41.0), compared to 55.3% (45.5–68.5), 1.9% (0.1–7.9) and 42.1% (27.8–52.4) using a composite signature selected using a combination of KW-H and DFA, or 82.0% (69.7–93.8), 8.2% (0.7–22.7) and 7.3% (0.7–21.0) using a fingerprint selected using KW-H and PCCA. The root mean square difference between the apportionment results using the fingerprints identified on the basis of the three different statistical approaches ranged from 5.5% to 25.7%, highlighting the sensitivity of source estimates to the tracers used. Regardless, the different composite signatures all suggested that unpaved recreational roads were the dominant source of the suspended sediment samples, underscoring the need for mitigation measures targeting these anthropogenic features of the catchment system, including closure to permit re-vegetation, surface ripping and/or mulching to improve infiltration or gravel re-surfacing to reduce exposure of bare surfaces to sediment mobilisation.

## Highlights

• Fingerprinting was used to assess the contribution of recreational roads to sediment yield.
• Three statistical approaches were used to select composite fingerprints.
• The Modified MixSIR Bayesian model was used for source apportionment.
• The source apportionment estimates were sensitive to the tracers.
• Recreational roads were the dominant source of suspended sediment.

## 1 Introduction

Anthropogenic disturbance associated with land development such as intensive farming for crops or livestock, deforestation or urbanisation, generally modifies catchment hydrology and increases soil erosion and catchment sediment yields (Foley et al., 2005, Seutloali and Beckedahl, 2015). Accelerated soil erosion and sediment delivery are identified as being of priority concern around the world because of many negative on-site and off-site consequences. On-site impacts include a reduction in soil productivity, whilst off-site effects include sedimentation of reservoirs or deterioration of water quality. Effective conservative actions for mitigating these effects require reliable information on key erosion processes and sediment sources at landscape scale.

One of the most substantial impacts on catchment erosion concerns road construction. Road networks artificially increase connectivity for transferring eroded materials through catchment systems (Croke et al., 1999, Motha et al., 2004). During construction and utilization, roads can accelerate soil erosion through diverse mechanisms such as removal of the protective vegetation cover and physical disturbance (Cao et al., 2015, Jimenez et al., 2013). Compaction of soil in road surfaces can reduce infiltration and as a result, runoff potential is increased (Pereira et al., 2015). Cut slopes formed in conjunction with road construction can increase mass movements and release considerable volumes of sediment (Doten et al., 2006, Wu et al., 2008). Some researchers also report switches in the types of erosion, such as to inter-rill and gully erosion, as a result of road construction (Clarke and Walsh, 2006, Imwangana et al., 2014). Megahan et al. (2001) investigated sediment production from forest road cutslopes in Idaho, USA and reported that erosion rates for the first winter period after construction averaged about five times greater than the average rates for subsequent seasons.

Among different types of roads, those that are unpaved or unsealed/unmetalled, tend to have the most significant contributions to sediment yield (Motha et al., 2004, Thomaz et al., 2014). In general, unpaved roads generate substantial amounts of sediment due to accelerated in situ erosion of unmetalled surfaces, destabilisation of side-cast material and the extension of the stream network and associated increased connectivity (Galia et al., 2017). Accordingly, much previous research has reported the importance of unpaved roads as major sediment sources in rural catchments (Bravo-Linares et al., 2017, Froehlich, 1995, Froehlich and Walling, 1992, Froehlich and Walling, 1997, Hoover, 1952, Ramos-Scharrón, 2018, Sheridan and Noske, 2007, Swift, 1984, Wemple et al., 2001, Ziegler and Giambelluca, 1997, Ziegler et al., 2000). Unpaved roads and verges are highly susceptible to hydraulic erosion processes and may produce significant amounts of sediment despite their relatively small areal extent (Collins et al., 2010a, Cooper et al., 2015, Ramos-Scharrón and MacDonald, 2007a, Russell et al., 2001).

Soil loss is closely related to erosivity (rainfall amount and intensity) and erodibility (resistance of the soil to both detachment and transport). The soil erodibility depends on topographic position, slope steepness, soil texture, aggregate stability, shear strength, infiltration capacity, organic and chemical content and land use management. The transformation of natural hillslope profiles, the interception of surface and subsurface flows, construction of road banks, reduced plant cover, and the compaction of soil on the road bed are all potential causes of changes in erodibility (Jordán-López et al., 2009). A range of factors can control sediment mobilisation from unsealed roads including, surface characteristics (Burroughs and King, 1989), road construction and maintenance (Elliot et al., 1999), area and slope (Sheridan et al., 2003), rainfall amount or intensity (Araujo et al., 2014, Megahan et al., 1991) and detachment by vehicle traffic (MacDonald et al., 2001, Reid and Dunne, 1984). Road traffic encourages sediment production by forcing fine sediment to the surface and via abrasion and crushing (Luce and Black, 1999, Sheridan et al., 2006, Ziegler et al., 2001). Here, ATV and dirt bike impacts can be as substantial as those resulting from regular truck traffic (Welsh, 2008). Unpaved roads can also act as secondary sediment sources as a result of deposition and subsequent remobilisation (Froehlich and Walling, 1992, Froehlich and Walling, 1997, Gruszowski et al., 2003, Wemple et al., 2001). Even well-designed road systems can alter catchment sediment budgets (Gucinski et al., 2001).

Unpaved recreational roads have received less attention from previous research than those serving commercial forestry but can, nevertheless, play an important role in accelerating soil erosion and sediment production, especially in hilly or mountainous terrains. Kidd et al. (2014), for example, studied the effect of such roads in Southwestern Virginia, USA, earmarking the role of recreational road stream crossings impacting on sediment delivery and water quality. Recreational roads can expose soils to higher rates of erosion in conjunction with exposure of the road bed, cutslopes, fill slopes, and (or) ditches (Spinelli and Marchi, 1996). Arnaez et al. (2004), for example, found that the cutslope of a road exhibited the highest erosion rates, attributing the losses to mass wasting and freeze-thaw processes along the cut banks which continuously release loose material. However, in contrast, Reid and Dunne (1984), reported that the cutslope, fill slope, and ditches of unpaved roads contributed only a small amount of sediment compared to the exposed road surface.

Identifying the relative contributions of sediment from unpaved recreational roads can be used to help inform erosion mitigation strategies. Historically, different techniques and methods have been used to identify and apportion sediment sources, ranging from traditional techniques such as erosion pins or field surveys of erosion features to sediment source fingerprinting (Collins and Walling, 2004). Some research has also applied modelling (Fu et al., 2010, Penna et al., 2014), a combination of direct volumetric measurements and ^137^Cs-based sediment budgeting (Katz et al., 2014), or high resolution topographic mapping (Tarolli et al., 2012, Tarolli et al., 2013). The concept of sediment fingerprinting refers to a field based technique that apportions or un-mixes, sampled sediment into distinguishable sources through the use of different tracers combined in a so-called composite fingerprint or signature (Collins et al., 1997, Collins et al., 2017, Owens et al., 2017, Walling et al., 1993). The importance of unpaved road margins in agricultural catchments and of unpaved roads in forested areas has already been explored using the source fingerprinting approach (see for example, Collins et al., 2010b, Haddadchi et al., 2013, Wallbrink et al., 2002). In contrast, the fingerprinting approach has not been used to investigate sediment contributions from unpaved recreational roads in a mountainous environment. The main objective of this study was therefore to use a composite fingerprinting method combining different statistical tests for source discrimination and a Bayesian un-mixing model for apportionment, to determine the relative importance of unpaved recreational roads in the Koohsar catchment, northern Tehran, Iran. It was hypothesized that unpaved recreational roads are the primary sediment source in the study catchment.

## 2 Materials and methods

### 2.1 Study area

**Fig. 1:**
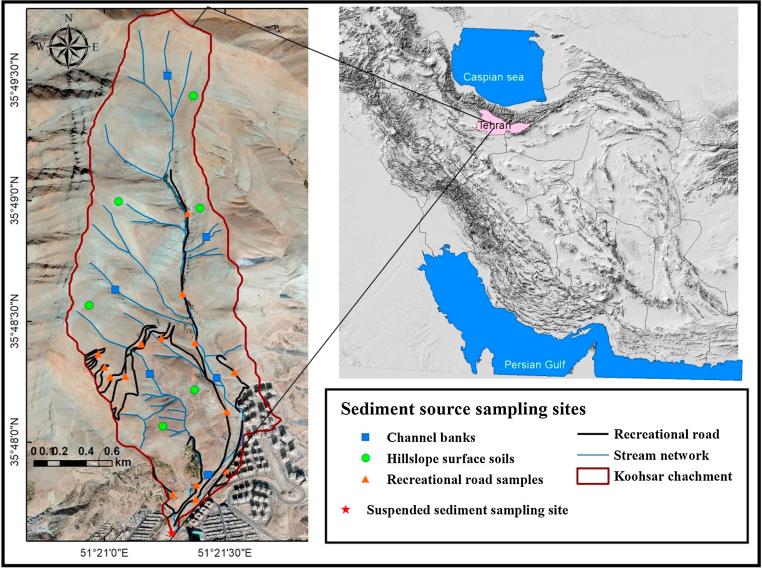
Map of the Koohsar study catchment and sampling sites.

The Koohsar catchment (292 ha) is located to the north of the capital of Iran, Tehran city, between 51⁰ 20′ 50″E to 51⁰ 21′ 45″E longitude and 35⁰ 47′ 36″N to 35⁰ 49′ 47″N latitude (Fig. 1) in the Southern Alborz Mountains. The topography of the Koohsar catchment is mountainous, with elevations ranging from 1721 to 2793 m, with a mean of 2154 m above sea level. The average slope gradient is 43.3%. The longest stream length is 4012 m. Land cover comprises 97.4% grazing land (284.5 ha) and 2.6% residential urban use (7.5 ha).

The catchment lithology is primarily Triassic sedimentary deposits including the Karaj Formation consisting of well-bedded green tuff and tuffaceous shale (EK). The soil orders within the catchment are mainly Entisols and Inceptisols. Long-term (20 years) mean annual precipitation at the Darakeh station near the study area is ca. 450 mm. In the upper parts of the region, precipitation is mostly snow.

**Fig. 2:**
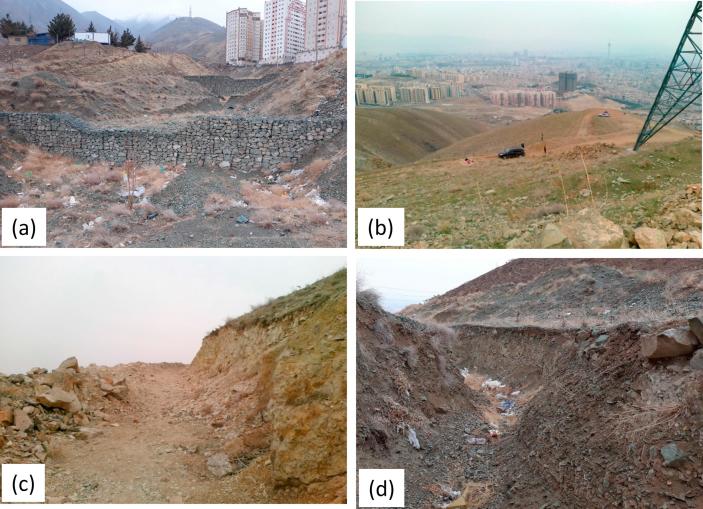
Photographs showing: (a) the consecutive check dams at the study catchment outlet; (b) rangelands on hillslopes; (c) an unpaved recreational road, and; (d) channel banks - b, c, d represent the key sediment sources in the study area.

Urban sprawl has the potential to influence geomorphic systems. Throughout the mountainous terrain in northern Tehran city and particularly in the areas immediately adjacent to the residential developments and associated dense populations, the construction of unpaved roads is widespread for the purpose of recreation. In the study area, several unpaved recreational roads have been constructed on hillslopes. These recreational roads are usually 3–6 m in width and often follow watersheds on hillslopes and along mountain ridges. The total length and drainage density of unpaved recreational roads in the study area are ca. 12.2 km and 4.2 km km^−2^, respectively. The road slope varies from 1.7% to 14%. The damage caused by the frequent use of these roads, and particularly by vehicle wheels promotes deepening. These unpaved recreational roads can increase runoff and erosion and should therefore be a major source of sediment; as a result, consecutive check dams have been constructed at the study catchment outlet to intercept high sediment loads (Fig. 2a).

### 2.2 Field sampling

*Sediment source samples:* Prior to sampling, field surveys were undertaken to identify potential sediment sources across the study area. Potential sediment sources were identified on the basis of soil erosion types observed within the study catchment: surface soil erosion on hillslope rangelands or unpaved recreational roads, and subsurface erosion affecting stream channel banks (Fig. 1, Fig. 2). A total of 27 source samples were collected to represent these key sediment sources, comprising six from the hillslopes, fifteen from unpaved recreational roads and six from channel banks. In order to increase the representativeness of the individual source samples, each surface (0–2 cm depth) sample for recreational roads (i.e. the road bed) or the rangeland hillslopes (0–5 cm depth) comprised a composite of five sub-samples collected within ca. 40 m^2^ at a specific site, whereas each subsoil (the full vertical extent of actively eroding profile faces) sample comprised a composite of 10 sub-samples collected within a ∼20 m long reach (interval 2 m) at each sampling site. The source samples were assumed to provide a spatially representative snapshot of erosion source conditions at the time of sample collection but the temporal representativeness of the source samples was not investigated explicitly. Each composite source sample weighed at least 1 kg. All source samples were retrieved from the field between May 2nd and May 5th 2016. Similarly to many previous studies, source samples were collected only once during the study period (Collins et al., 2017).

*Suspended sediment samples:* Although a range of target sediment sample types can be used (Collins et al., 2017, Owens et al., 2017, Walling, 2013, Walling and Collins, 2016), the majority of sediment fingerprinting studies rely on the collection and analysis of suspended sediment transported during flood events (Collins and Walling, 2004, Devereux et al., 2010, Mizugaki et al., 2008, Mukundan et al., 2012, Pulley et al., 2015). In this study, five bulk suspended sediment samples were collected from the study catchment outlet. These samples spanned one water year to capture potential seasonal contrasts in sediment dynamics. All individual suspended sediment samples were retrieved during flood events by collecting a large volume (40 to 80 L) of water manually at varying time intervals during five rainfall-runoff events (December 12, 2015, January 15, 2016, March 01, 2016, March 22, 2016 and April 25, 2016) at the catchment outlet. These samples were decanted and manually filtered to de-water sufficient sediment mass for all laboratory analyses. The five rainfall-runoff events sampled for sediment were monitored for discharge. Discharges were measured manually using the velocity-area method (Gordon et al., 2004) in a rectangular structure at the study catchment outlet. This method requires measurement of the area of the channel cross section and the average stream velocity during each rainfall-runoff event. Discharge is then calculated as Q = V × S: where Q is discharge (m^3^ s^−1^), V is average velocity (m s^−1^) and S is cross-sectional area of the water (m^2^). Area was calculated from cross-section measurements (calculated by multiplying width and depth). Flow velocity was measured with a current meter (OTT Hydromet, Germany). As the width of the rectangular structure at the study catchment outlet was fixed (constant), the velocity and depth of flow were measured three times during each storm event. The mean (estimated using three measurements for each individual event) discharge during the runoff events sampled on December 12th 2015, January 15th 2016, March 01st 2016, March 22nd 2016 and April 25th 2016 was estimated (minimum-maximum ranges in brackets) at: 1.53 (1.19–1.90), 2.14 (1.28–3.15), 1.89 (0.96–2.75), 1.17 (0.67–1.64) and 1.75 (0.80–2.36) m^3^ s^−1^, respectively. A snapshot of suspended sediment concentration during each event was measured by collecting an instantaneous 1.5 L sample of runoff water and using manual gravitational filtering and weighing in the laboratory. The sediment concentrations during the events sampled on December 12th 2015, January 15th 2016, March 01st 2016, March 22nd 2016 and April 25th 2016 were estimated at 52, 124, 96, 181 and 172 mg L^−1^, respectively. Given that the work reported here was a preliminary investigation of the importance of recreational roads as a sediment source in the study catchment, the experimental design did not explore the implications of spatially heterogeneous snowfall or varying proportions of rainfall/snowfall or freeze-thaw on the magnitude, timing and intensity of runoff induced erosion processes and thereby the estimated source proportions. Instead, the work herein provided a first pass assessment of the relative contribution of recreational road erosion to sediment export from the study area.

### 2.3 Laboratory measurements of tracers

Dry sieving revealed that the <63 µm fraction was most representative of the suspended sediment samples collected in this study. Consequently, only the <63 µm fraction of the sediment and source samples was used for the analysis and comparison of fingerprint properties. In order to measure the concentrations of geochemical tracers, one gram of the sediment and source samples (<63 µm) was digested in aqua regia (HCl–HNO3; 3:1) using a Velp Thermo-reactor at 95 °C for two hours. After filtering the extracts through S&S ME24 (0.2 µm) filter papers, the solutions were analysed by a Varian SpectrAA-20 Plus calibrated using an element standard solution (Merck KGaA, Frankfurter, Germany) for Ca, Co, Fe, K, Mg, Mn, Na, Pb, Sr, and Zn concentrations. The results showed that analytical error was less than 5% for all elements. Total organic carbon content was measured by the Walkley-Black method (Skjemstad and Baldock, 2008).

### 2.4 Tracer conservation tests

A range of factors can influence tracer conservation in the natural environment, including redox, temperature, selective particle transport, adsorption/desorption or precipitation/dissolution (Stumm and Morgan, 1996). The complex interplay of these controls will be highly variable and site-specific. A three-part procedure was used to assess tracer conservation. Firstly, a standard bracket or range test (Foster and Lees, 2000) was used to identify non-conservative tracers, whereby the tracer concentrations in the suspended sediment samples were compared with the corresponding minimum and maximum ranges associated with the sources samples, which bound the un-mixing polygon (Zhang and Liu, 2016). This standard test does not provide truly definitive confirmation of tracer conservation, but instead, is used to confirm that major transformation is not occurring during sediment mobilisation and delivery. Secondly, in addition to the standard test, the tracers were checked using a stricter test whereby the sediment sample means should fall within the corresponding source means rather than their full ranges. This stricter range test is useful because sediment sample tracer concentrations commonly exhibit limited variation compared with source samples meaning that tracers easily pass the standard range test procedure. Thirdly, to augment the testing for conservatism further, biplots of tracers included in the final statistically-verified composite fingerprints were also used to compare source and sediment samples. Here, the source and sediment sample values will plot in the same space or along the same line if tracer behaviour is conservative. In combination, these three tests provide a more robust assessment of conservatism than the standard range test alone.

### 2.5 Statistical discrimination of sediment sources

The statistical analysis employed to identify different composite fingerprints for discriminating between the potential sediment sources used three approaches: (1) the Kruskal–Wallis H-test (KW-H), (2) a combination of the KW-H as step one and discriminant function analysis (DFA) as step two, and (3) a combination of the KW-H as step one and principal component & classification analysis (PCCA) as the second step. Three final composite signatures were therefore selected on this basis. All statistical analyses were performed using STATISTICA V.8.0 (StatSoft, 2008). It is well-established that selecting differing composite signatures using independent statistical tests based on different rules can generate contrasting estimates of source apportionment. This reflects the sensitivity of the source fingerprinting approach to the tracers included in any composite signature. The international tracing community has widely accepted that it is better to be explicit about this aspect of sensitivity by using more than one composite signature selected by different statistical tests (e.g., Collins et al., 2012, Collins et al., 2013, Collins et al., 2014, Collins et al., 2017, Owens et al., 2017, Palazón et al., 2015, Palazón and Navas, 2017).

#### 2.5.1 Kruskal–Wallis H-test

The KW-H is a non-parametric equivalent of one-way ANOVA to compare more than two groups, and tests the null hypothesis that the different groups in the comparison are drawn from the same distribution or from distributions with the same median. However, unlike one-way ANOVA, it does not make assumptions about homogeneity of variance or normal distributions. Thus, the interpretation of the KW-H is basically similar to that of parametric one-way ANOVA, except that it is based on ranks rather than means (Dytham, 2011).

#### 2.5.2 Discriminant function analysis (DFA)

Those tracers exhibiting statistically significant differences between the potential sediment sources, using KW-H, were included in the DFA. DFA has been used extensively in sediment source fingerprinting investigations (e. g. Gellis and Noe, 2013, Laceby et al., 2015, Walling, 2013) since an early study by Collins et al. (1997). The basis of DFA is to provide a set of weightings that allow the source groups to be distinguished. The weightings can then be used on individuals that are not assigned to a group to provide a probability of them belonging to each of the possible source groups. Different tests including eigenvalue, canonical correlation, Wilks' lambda, and squared Mahalanobis were used to determine whether the discriminant functions were statistically significant. Membership of the sediment source groups was the dependent variable, whereas the measured tracers constituted the independent variables.

#### 2.5.3 Principal component & classification analysis (PCCA)

PCCA can be used as a classification technique in addition to reducing the dimensions of the original variable space so that the relations among variables and cases can be highlighted. To do this, the variables and the cases are plotted in the space generated by the principal component axes. This technique works in very much the same way as PCA but with one crucial difference; the individual samples must be assigned to source groups before the analysis. The test then calculates the variable weightings that will maximize the differences between source groups rather than individuals as is the case with PCA. The PCCA produces weightings that will allow you to identify those variables that are the most different between source groups and discard those that are the same.

Only those tracers with significant differences between the potential sediment sources, using KW-H, were included in the PCCA. Principal components with eigenvalues >1 were retained and subjected to a varimax rotation to minimize the number of tracers that have high loadings on each PC. Under a particular PC, each tracer is given a weight or PC loading that represents the contribution of that tracer to the composition of the PC. Only the highly-weighted tracers were retained from each PC. Highly-weighted tracer loadings were defined as having absolute values within 10% of the highest tracer loading. When more than one tracer was retained under a single PC, multivariate correlation coefficients were employed to determine if the tracers could be considered redundant and, therefore, eliminated from the final set of tracers (i.e. composite fingerprint). If the highly-weighted tracers were not correlated (assumed to be a correlation coefficient <0.60) then each was considered important, and thus, retained in the final composite signature. Among well-correlated tracers, the tracer with the highest PC loading (absolute value) was chosen for the final composite fingerprint. Once the composite signature was chosen, a final check was undertaken to identify significant differences among the potential sediment sources based on the PC scores of each sample using one-way ANOVA (F-test) and Tukey HSD post-hoc tests (P < 0.05).

### 2.6 Source apportionment using the Modified MixSIR Bayesian un-mixing model

Some recent sediment source tracing studies applying un-mixing models have used the Modified MixSIR Bayesian model (Nosrati et al., 2014, Nosrati et al., 2018). This model provides a Bayesian rather than frequentist (e.g. Collins et al., 1997, Walling et al., 2006, Walling et al., 2008) approach to apportionment modelling and builds upon earlier tools constructed for isotopic studies (e.g. Moore and Semmens, 2008, Parnell and Jackson, 2011, Parnell et al., 2010).

The Modified MixSIR Bayesian statistical approach quantifies the relative contributions of sediment from different sources by calculating probability distributions for the proportional contribution (f_i_) of each source i to the downstream target sediment samples in three stages: 1) determination of the prior probability distributions for model parameters, 2) construction of a likelihood function for the statistical model, and 3) derivation of the posterior probability distributions for the parameters using the Bayes rule to adjust the prior distribution based on the observed data. The Bayes rule states that the posterior probability distribution for all f_i_ is proportional to the prior probability distributions multiplied by the likelihood, and then dividing by their sum, viz.:

(1) $$P(f_{q}\left|\mathrm{data})=\frac{L(data\left|f_{q})\times p\left(f_{q}\right)\right)}{\sum L(data\left|f_{q})\times p\left(f_{q}\right)\right)}\right)$$

where L(data|fq) is the likelihood of the data given f_q_, p(f_q_) representing the prior probability being true, based on prior information, and f_q_ is the proportional source contributions of q proposed vectors.

The relative contributions of sediment are factored into the model by defining mean and variance parameters for each sediment source i and the final sets of tracers (composite fingerprints; j). Modelling source contributions using more than one composite signature permits an assessment of the potential uncertainty resulting from different fingerprint property sets (Collins et al., 2012).

The proposed tracer distributions for the target sediment mixtures collected from the study catchment outlet are determined by solving for the proposed means${\hat{\mu }}_{j}$and standard deviations${\hat{\sigma }}_{j}$of the sediment mixtures based on the randomly drawn f_i_ values comprising a vector f_q_:

(2) $${\hat{\mu }}_{j}=\sum_{i=1}^{n}\left(f_{i}\times m_{j_{\mathrm{Sourc}e_{i}}}\right)$$

(3) $${\hat{\sigma }}_{j}=\sqrt{\sum_{i=1}^{n}\left(f_{i}^{2}\times S_{j_{\mathrm{Sourc}e_{i}}}^{2}\right)}$$

where $m_{j_{\mathrm{Sourc}e_{i}}}$ in Eq. (3) is the mean and $S_{j_{\mathrm{Sourc}e_{i}}}^{2}$ in Eq. (4) is the variance of the jth sediment tracer and the ith sediment source.

Based on the ${\hat{\mu }}_{j}$ and ${\hat{\sigma }}_{j}$of each property comprising each final composite fingerprint, the likelihood of the data given the proposed sediment mixture is calculated as:

(4) $$L\left(x\left|{\hat{\mu }}_{j}\right),{\hat{\sigma }}_{j}\right)=\prod_{k=1}^{n}\prod_{j=1}^{n}\left[\frac{1}{{\hat{\sigma }}_{j}\times \sqrt{2\times \pi }}\times \exp\left(-\frac{{\left(X_{\mathrm{kj}}-{\hat{\mu }}_{j}\right)}^{2}}{2\times {\hat{\sigma }}_{j}^{2}}\right)\right]$$

where $X_{\mathrm{kj}}$represents the *j*th tracer property of the *k*th sediment sample.

Using a version of the sampling-importance-resampling (SIR) algorithm (Moore and Semmens, 2008), we generated 10^6^ samples from the posterior distribution of the estimated target sediment mixtures. This method establishes a threshold acceptance value prior to sampling and uses it simultaneously to resample, as the un-normalized posterior probabilities for each fq sample are calculated.

## 3 Results and discussion

### 3.1 Final tracers and composite fingerprints for discriminating the potential sediment sources

Table 1 compares the tracer concentrations in the sediment sources and five suspended sediment samples collected at the study catchment outlet. In addition, Table 1 also presents the results of the normality test for tracers, showing that all measured tracers had normal distributions (a prerequisite for using the tracers in a Bayesian model). The results of the standard bracket test showed that all tracers were generally conservative. In addition to the standard test, the results of comparing the sediment means with the corresponding source means showed that all tracers except K and Na are conservative (Table 1). Therefore, these two tracers were removed from further analysis.

**Table 1:** Tracer concentration data for the sediment sources and suspended sediment samples, the results of the normality test and the Kruskal-Wallis H-test results for discriminating the sediment sources.

Sediment sources		Tracers										
		Ca (mg Kg^−1^)	Co (mg Kg^−1^)	Fe (mg Kg^−1^)	K (mg Kg^−1^)	Mg (mg Kg^−1^)	Mn (mg Kg^−1^)	Na (mg Kg^−1^)	OC (g Kg^−1^)	Pb (mg Kg^−1^)	Sr (mg Kg^−1^)	Zn (mg Kg^−1^)
Recreational road	Mean	17409.5	8.8	27250.5	1634.8	6746.6	781.2	1884.0	3.0	47.8	229.4	69.3
	SD	3946.7	1.0	2189.4	200.2	559.8	56.6	763.5	1.5	13.9	27.2	3.8
Hillslope surface soils	Mean	9162.6	8.6	24649.4	1240.4	4887.9	705.8	2099.9	1.8	53.1	161.4	65.9
	SD	3572.4	1.3	2885.7	194.4	330.8	25.9	440.1	0.7	15.6	2.1	2.7
Channel banks	Mean	10940.3	6.1	24344.9	1321.1	4253.8	639.5	2474.5	4.8	59.1	149.5	72.1
	SD	4518.6	1.6	721.5	373.6	269.8	40.5	199.3	1.8	13.3	3.5	6.9
Kolmogorov-Smirnov test for normality	K-S d	0.14	0.24	0.11	0.07	0.15	0.14	0.13	0.15	0.10	0.2	0.09
	p-value	0.16	0.07	0.2	0.2	0.10	0.16	0.2	0.10	0.2	0.06	0.2
KW-H test^2^	Chi-Square	13.6	9.6	9.5	n.c.^1^	20.3	17.6	n.c.	7.6	2.6	21.0	5.5
	p-value	0.001*	0.008*	0.009*	–	<0.0001*	0.0002*	–	0.02*	0.27	<0.0001*	0.07

*Suspended sediment samples*												
S-1		16224.3	8.0	26239.1	1012.3	5256.2	661.6	1735.1	3.0	40.2	229.1	67.8
S-2		22742.8	6.4	24008.4	1240.4	5328.7	661.6	1897.9	2.1	30.8	230.2	68.9
S-3		17129.5	8.0	26316.1	1052.9	6560.6	604.8	849.1	6.3	58.4	178.4	62.7
S-4		16816.9	8.0	24393.0	1131.0	5546.1	699.5	1871.9	0.8	62.9	230.2	69.9
S-5		15631.7	4.8	23335.3	1162.3	5220.0	680.6	1813.3	2.1	53.8	237.0	67.4
Mean		17709.0	7.0	24858.4	1119.8	5582.3	661.6	1633.4	2.9	49.2	221.0	67.3

1 Non-conservative.

2 KW-H test, Kruskal-Wallis H-test.

* Critical p-value = 0.05.

Table 1 also shows the results of applying the KW-H test which indicated that seven tracers (Ca, Co, Fe, Mg, Mn, OC and Sr) exhibited a statistically significant difference between the three potential sediment sources. Those tracers (Pb and Zn) unable to discriminate the potential sources were discarded from further analysis.

The seven tracers selected by the KW-H test were entered into the stepwise DFA (Table 2). The largest eigenvalue of the first function (25.1) corresponds to the eigenvector in the direction of the maximum spread of the groups’ means. The Wilk’s lambda value of the first function (0.015) indicated that 98.5% of the total variance among the potential sediment sources was explained by these tracers. The canonical correlation value was 0.98 and indicated a strong correlation between the discriminant scores and the individual source groups.

**Table 2:** Summary of the backward DFA.

DFA parameters	Result
*Function 1*	
Eigenvalue	25.1
Wilks' lambda	0.015
Canonical correlation	0.98
*Function 2*	
Eigen value	1.5
Wilks' lambda	0.39
Canonical correlation	0.78

*Sediment source samples classified correctly (%)*	
Recreational road	100.0
Hillslope surface soils	100.0
Channel banks	100.0
Total	100.0

*Sampling sites of sediment sources assigned by DFA*	
Recreational road	15.0
Hillslope surface soils	6.0
Channel banks	6.0

*Squared Mahalanobis distance*	
Recreational road × Hillslope surface soils	52.8
Recreational road × Channel banks	126.7
Hillslope surface soils × Channel banks	29.7

*Squared Mahalanobis F-value*	
Recreational road × Hillslope surface soils	49.5*
Recreational road × Channel banks	118.8*
Hillslope surface soils × Channel banks	19.5*

* Significant at 0.01 level.

**Fig. 3:**
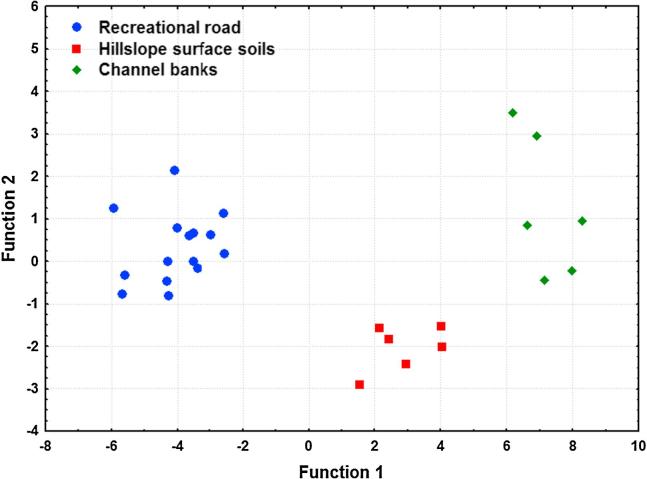
Scatterplot of the first and second discriminant functions calculated using backward DFA associated with selection of the composite signature comprising Ca, Mg, Mn, and OC.

The squared Mahalanobis distance showed that the sediment sources were well separated by the shortlisted tracers (Table 2). The backward stepwise DFA yielded classification matrices assigning 100% of the cases (i.e., source samples) to the correct groups (Table 2). Stepwise selection using Wilks’ lambda indicated that a composite signature comprising four tracers (Ca, Mg, Mn and OC) provided significant discriminatory power on the basis of the DFA model (Table 3). The results of different tests within DFA indicated that the discriminatory power of Mg and Mn is perfect (Table 3). Partial Wilks’ lambda is the Wilks’ lambda for the unique contribution of the respective tracer to the discrimination between individual source groups. The smaller the Partial Wilks’ lambda, the greater the contribution to the overall discrimination. The Partial Wilks’ lambda values suggested that Mg contributed the most, Mn second most, OC third most and Ca the least to the overall discrimination (Table 3). A scatterplot using the first and second discriminant functions calculated using backward DFA confirmed that the samples collected to characterise the different potential sediment sources were well separated (Fig. 3).

**Table 3:** Final outputs of the stepwise backward DFA.

Tracer	Wilks’ lambda	Partial Wilks’ lambda	F-remove	p-level	Tolerance
Ca	0.03	0.54	8.9	0.002	0.51
Mg	0.15	0.10	90.1	<0.001	0.45
Mn	0.05	0.29	26.2	<0.001	0.36
OC	0.03	0.52	9.7	0.001	0.70

Tracers passing the KW-H test (Ca, Co, Fe, Mg, Mn, OC and Sr) were also tested using PCCA. All tracers were further explored as an alternative means of reducing the number of tracers and problems of multicollinearity. The results of PCCA showed that the first three principal components (PCs) with eigenvalues >1 accounted for >86% of the variability among the tracer values for the source groups (Table 4). The PC corresponding to the largest eigenvalue (3.7) accounted for approximately 52% of the total variance. The second PC corresponding to the second eigenvalue (1.4) accounted for approximately 19% of the total variance (Table 4). The PC corresponding to the smallest selected eigenvalue (1.0) accounted for approximately 14% of the total variance (Table 4).

**Table 4:** PCCA factor coordinates of the variables and the eigenvalues of the correlation matrix.

Tracer	PC 1	PC 2	PC 3	Communalities
Ca	0.79	0.37	0.32	0.87
Co	0.51	−0.54	−0.57	0.88
Fe	0.55	−0.67	0.22	0.79
Mg	0.89	−0.37	0.08	0.93
Mn	0.82	0.34	0.03	0.80
OC	−0.42	−0.42	0.69	0.83
Sr	0.93	0.24	0.11	0.93
Eigenvalue	3.7	1.4	1.0	
% Total variance	52.9	19.4	13.9	
Cumulative % variance	52.9	72.3	86.2	

*Mean scores of the three sediment sources*				
Recreational road	−1.36 a^1^	−0.13 a	−0.19 a	
Hillslope surface soils	−0.62b	0.12 a	−1.29b	
Channel banks	0.79c	0.79b	0.81 a	

*ANOVA results*				
F-value	92.1	11.3	15.2	
p-value	<0.0001	<0.001	<0.001	

1 Different small letters indicate that scores are significantly different at the 5% level, based on the Tukey HSD Post Hoc test.

**Fig. 4:**
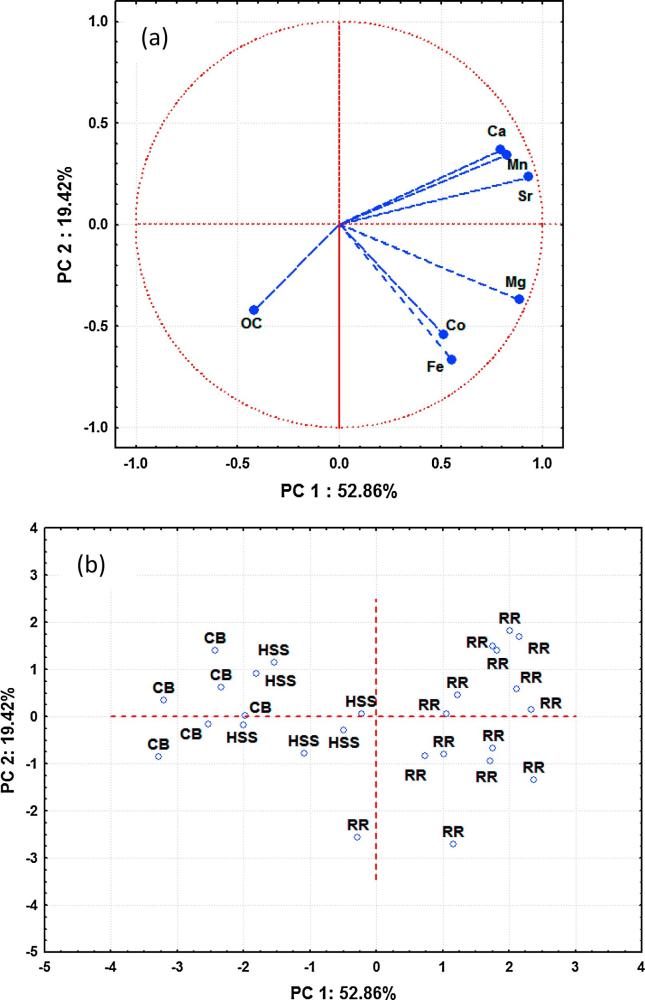
(a) Projection of the optimum composite tracers on the PC-plane using PCCA, (b) Projection of the cases on the PC-plane using PCCA; RR: recreational road; CB: channel banks; HSS: hillslope surface soils.

The highly-weighted tracers under PC1 with absolute values within 10% of the highest tracer (0.93 value for Sr) loading (the loading of selected tracers should be larger than 0.84) were Mg and Sr. Only Sr was retained for the final composite signature because Mg and Sr were strongly correlated (r = 0.76). Also, this tracer was most important due to the highest communality estimate (Table 4). Under PC2, the highly-weighted tracer with absolute values within 10% of the highest tracer (0.67 value for Fe) loading (the loading of selected tracers should be larger than 0.60) was Fe. Under PC3, the highly-weighted tracer (0.69 value for OC) with absolute values within 10% of the highest tracer loading (the loading of selected tracers should be larger than 0.62) was OC. These results selected these three tracers (Sr, Fe and OC) as an alternative composite fingerprint on the basis of the PCCA model (Table 4). The plot of principal component (PC) coordinates of tracers for the first two PCs showed that the three selected tracers were represented by the current set of PCs (Fig. 4a). Thus, the set of selected tracers (i.e. composite fingerprint) clearly provided discrimination between the three potential sediment sources (Fig. 4b). These results illustrated that PCCA can be used as a tool for identifying important dimensions in a set of tracers and to identify those sediment sources with similar or dissimilar characteristics.

**Fig. 5:**
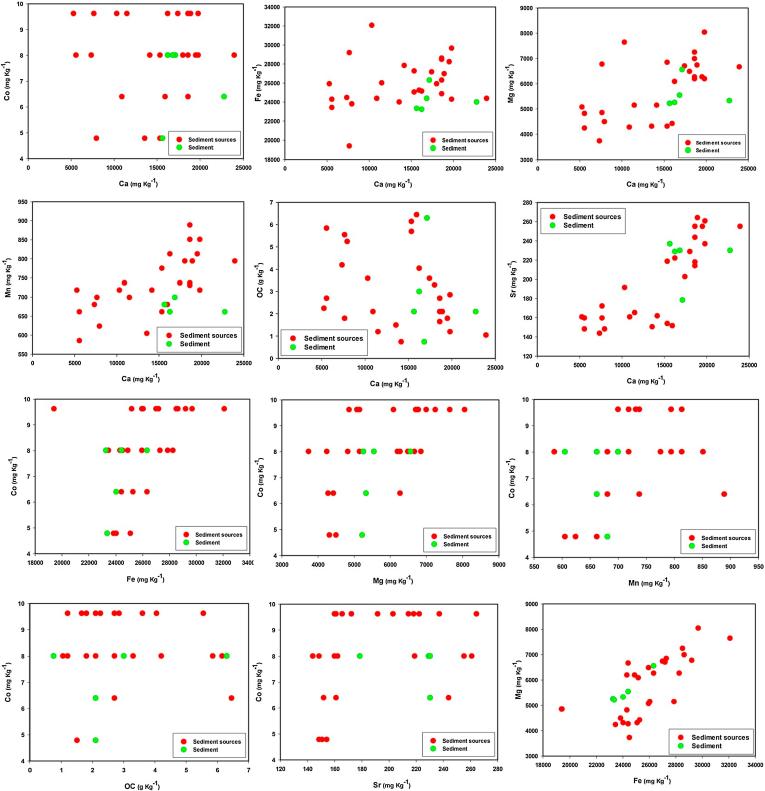

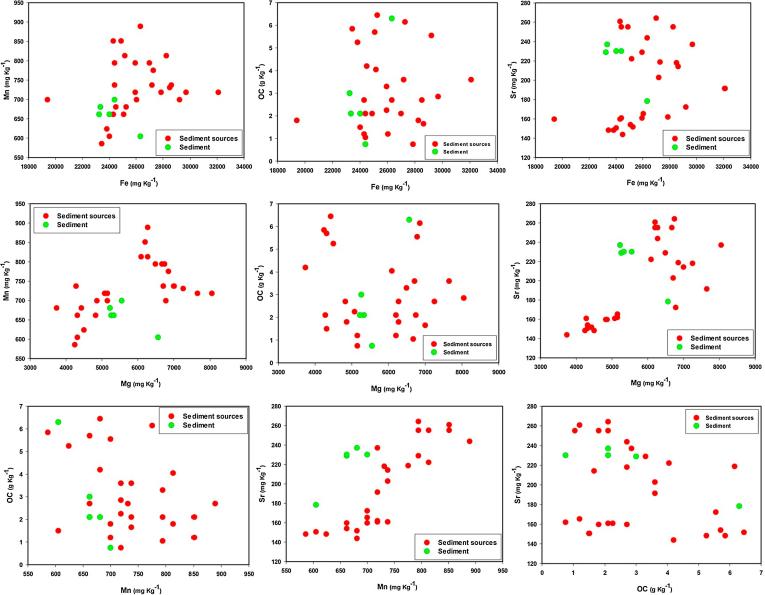
Biplots of all pairings for the tracers selected in the final composite signatures for discriminating and apportioning source contributions to sediment samples.

PCs scores were calculated using the resulting component score coefficient matrix and tested for significant differences between the potential sediment sources (Table 4). PC scores for both PCs varied significantly with sediment source (Table 4). Thus, the tracers related to these PCs provided a basis for selection of an alternative composite signature (Sr, Fe and OC). For the tracers selected in the final composite signatures, the biplots of all tracer pairings for source and sediment samples were compared. The results confirmed that there is no major tracer transformation (Fig. 5).

### 3.2 Sediment source contributions

**Fig. 6:**
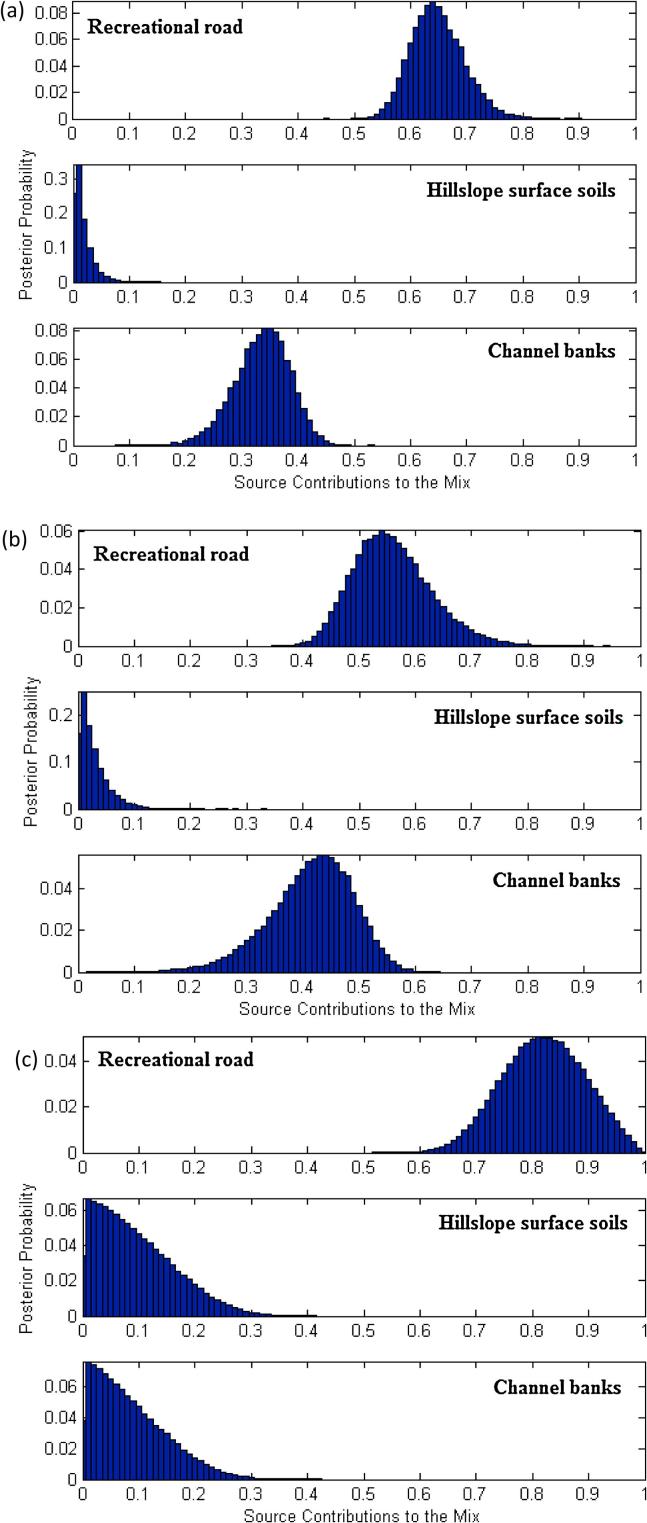
Probability density functions for the estimated source contributions using the final composite signatures selected by (a) KW-H, (b) a combination of KW-H as step one and discriminant function analysis (DFA) as step two, and (c) a combination of KW-H as step one and principal components & classification analysis (PCCA) as step two.

Using priors and estimates of uncertainty associated with the un-mixing model inputs, a Modified MixSIR model run of 10^6^ iterations resulted in convergence on the posterior contributions from the sources using the three different composite signatures selected using the independent tests (Fig. 6). Using KW-H (Table 5), the relative contributions (with corresponding uncertainty ranges) from recreational roads, hillslope surface soils and channel banks were estimated as 64.5% (57.7–73.1), 1.1% (0.1–4.9), and 33.9% (24.9–41.0), respectively. Using the alternative composite signature selected by a combination of KW-H and DFA (Table 5), the corresponding respective contributions and associated uncertainty ranges were estimated as 55.3% (45.5–68.5), 1.9% (0.1–7.9) and 42.1% (27.8–52.4). Finally, on the basis of the composite signature selected using a combination of KW-H and PCCA (Table 5), the relative contributions from recreational roads, hillslope surface soils and channel banks were estimated as 82.0% (69.7–93.8), 8.2% (0.7–22.7) and 7.3% (0.7–21.0), respectively. The root mean square difference (Table 5) between the estimated sediment contributions using the three different composite signatures ranged from 5.5% (hillslope surface soils) to 25.7% (channel banks). The predicted source contributions were therefore sensitive to the composite fingerprint used, underscoring the need to use multiple signatures when investigating sediment source contributions (cf. Collins et al., 2017, Nosrati et al., 2018, Owens et al., 2017, Palazón and Navas, 2017).

**Table 5:** Relative contributions from the sediment sources to the suspended sediment samples using composite signatures selected by different statistical approaches and corresponding root mean square differences between the source apportionment results.

Statistical approaches for selecting composite fingerprints	Sediment source		
	Recreational road (%)	Hillslope surface soils (%)	Channel banks (%)
KW-H (Tracers: Ca, Co, Fe, Mg, Mn, OC, Sr)	64.5 (57.7–73.1)*	1.1 (0.1–4.9)	33.9 (24.9–41.0)
Combination of KW-H and DFA (Tracers: Ca, Mg, Mn, OC)	555.3 (45.5–68.5)	01.9 (0.1–7.9)	442.1 (27.8–52.4)
Combination of KW-H and PCCA (Tracers: Fe, OC, Sr)	882.0 (69.7–93.8)	88.2 (0.7–22.7)	77.3 (0.7–21.0)
Root mean square difference	19.2	5.5	25.7

* The values in parentheses show the uncertainty ranges (90% confidence limits: 5%–95%).

### 3.3 Discussion

Previous studies in other areas of the world have highlighted unpaved roads as landscape features experiencing high erosion rates and as important contributors to sediment fluxes in river catchments. For example, Ramos-Scharrón and MacDonald, 2007a, Croke et al., 1999 observed erosion rates of between four and six orders of magnitude higher on unpaved roads than on undisturbed hillslope areas in the U.S. Virgin Islands, and coastal southeast Australia, respectively. Jordán-López et al. (2009) reported that highest soil loss rates were found on road banks and that the total soil loss on road banks was between 3 and 18 times higher than corresponding estimates for road beds and side cast fills, respectively. Megahan et al. (2001) reported that erosion rates for the first winter period after forest road construction in Idaho, USA, averaged about five times greater than the corresponding average rates for subsequent monitoring seasons. Motha et al. (2004) reported that relative contributions from gravel-surfaced roads, grouped lands (un-graveled roads, pasture lands and cultivated lands on basalt-derived soils), cultivated lands on granite-derived soils, and forest areas to sediment sampled on the falling limbs of event hydrographs were 41 ± 17%, 18 ± 13%, 13 ± 11% and 14 ± 7%, respectively. The relative contributions during a peak discharge event were 52 ± 12%, 30 ± 17%, 15 ± 11% and 17 ± 8%, respectively. Froehlich and Walling (1997) reported that the evidence provided by radiocaesium fingerprints suggested that the major source of the suspended sediment transported by the Homerka stream was unmetalled roads which occur throughout both the forested and the agricultural zones of this study basin in the Polish Flysch Carpathians. Equally, data measured using rainfall simulations on plots or on slope segments also demonstrated that unpaved roads are the major source of suspended sediment. Jordán-López et al. (2009) reported that the highest sediment concentration in runoff was detected on the roadbank, from which mean sediment yield was estimated at 13.7 g L^−1^. Ramos‐Scharrón and MacDonald (2007b) reviewed studies of small scale erosion on unsealed roads. The sediment concentrations they reported ranged from 70 mg L^−1^ to 227,000 mg L^−1^. Fu et al. (2010) reviewed road erosion data provided by rainfall simulator and flume studies. The hourly sediment erosion rates ranged from 0.3 to 35.9 g m^−2^ mm^−1^ h^−1^. These estimates suggest a very large range in road erosion rates across different study areas.

The source apportionment estimates generated here using different composite signatures underscored the importance of sediment loss from recreational roads in the study catchment. Unpaved recreational roads have a propensity to alter catchment hydrology and sediment dynamics, with common impacts including: increasing Hortonian overland flow (Ramos-Scharrón and LaFevor, 2016, Ziegler and Giambelluca, 1997); altering the magnitude and timing of peak flows in response to rainfall (La Marche and Lettenmaier, 2001); accelerating runoff responses to precipitation (Froehlich, 1995), and; increasing sediment yields (Bilby, 1985, Brunsden and Thornes, 1979, Fransen et al., 2001, Poeppl et al., 2017). Such hydro-sedimentological impacts largely reflect the role of unmetalled roads in increasing drainage density by artificially extending flow pathways and the stream network (Croke and Mockler, 2001, Luce and Wemple, 2001, Takken et al., 2008). Here, it is important to note, however, that the impact of unmetalled roads on sediment dynamics via increasing drainage density and connectivity from slopes to channels, depends on source strength associated with the erodibility of unmetalled road surfaces (Croke et al., 2005). In addition, the impact of unmetalled roads on hydro-sedimentological response is strongly influenced by the location and arrangement of road drainage structures including mitre drains and culverts (Croke et al., 2005). Gully development at culvert outlets enhances connectivity between unmetalled road networks and streams, with such gully development being controlled by contributing area, road runoff and hillslope gradient (Croke et al., 2005, Montgomery, 1994). Where runoff mobilising sediment from unpaved road networks discharges onto well-vegetated slopes, impacts on hydro-sedimentological responses are smaller (Haupt, 1959).

Given that the source apportionment estimates underscored the importance of unpaved recreational roads in the study area, appropriate mitigation measures need to be identified and implemented. Existing common ways of reducing the adverse impacts of unmetalled roads on catchment sediment dynamics include decommissioning or closing roads (Switalski et al., 2004, Weaver et al., 2015). Road closure, whilst not always reducing infiltration rates to background levels (Foltz et al., 2009), does improve infiltration, reduce surface degradation by vehicle traffic and permit re-vegetation, all of which, collectively reduce sediment production (Foltz et al., 2009). In situations where it is not possible to close or decommission unpaved recreational roads, alternative mitigation can comprise compaction treatment by roadbed ripping (Luce, 1997, Weaver et al., 2015), although here, the treatment effect can be transient (Robichaud, 2000). Furthermore, ripping alone has been shown to not be as effective as a combination of ripping and mulching (Sosa-Pérez and MacDonald, 2017). Where unpaved recreational roads are retained, gravel re-surfacing can be used to enhance trafficability and to reduce mobilisation of surface sediment from the road bed (Brown et al., 2013, Clinton and Vose, 2003).

### 3.4 Limitations

The source apportionment estimates discussed above must inevitably be interpreted in the context of some limitations. Numbers of samples collected to characterise individual sources by any sediment fingerprinting investigation are inevitably constrained by available budgets as well as practical considerations including those associated with the mountainous terrain of the study area and rarely, if ever, satisfy statistically-based probability sampling (Collins and Walling, 2004). A single source sampling campaign was undertaken and so any potential sensitivity issues surrounding general replicability associated with the timing and locations of the source samples including those from the unpaved recreational roads were not investigated explicitly. Different locations within the same source category will clearly be characterised by different erosion histories, spatial patterns and intensities meaning that repeat source sampling could affect signatures and hence the overall findings. Equally, the work, on account of its duration, did not consider the potential impacts of spatially heterogeneous snowfall, varying proportions of rainfall/snowfall or freeze-thaw on the magnitude, timing and intensity of runoff induced erosion processes and thereby potentially the source apportionment estimates. The source apportionment estimates are scale dependent and it important to recognise that they might differ for different sampling locations along the study catchment channel network (Koiter et al., 2013). In this study, target suspended sediment for source apportionment was collected from a single downstream location on the main stem of the study river. As a result, the source proportions pertain to this specific sampling site and additional stream network locations would need to be included to assess potential variations in sediment source contributions at different scales within the study area. Sediment sampling also needs to be temporally representative and this study sampled the catchment outlet across one water year, but in the context of hydro-climatic variability, it would be informative to sample additional water years. For this reason, some previous sediment source tracing investigations, albeit in different physiographical settings to the one involved here, have reported sampling spanning more than a single year (e.g. Walling et al., 2006, Walling et al., 2008), although sediment sample collection during a single year is also reported in published studies (e.g. Gellis and Noe, 2013, Nosrati, 2017). Tracer property transformation during mobilisation and delivery to, and through, the stream network, was assumed not to be significant enough to impact on the predicted source proportions. Here, although tracer properties were tested for major transformation using a three-part procedure, this does not confirm a complete absence of tracer transformation and this potential issue associated with source tracing requires further work. Various factors can influence sediment tracer conservation during mobilisation and transport through catchment systems including biogeochemical processes such as adsorption or desorption (Förstner and Salomons, 1980), as well as physical factors such as particle size selectivity (Grygar and Popelka, 2016, Horowitz, 1991). A limited amount of previous research has tested tracer conservation experimentally (e.g. Motha et al., 2002) and both past (Motha et al., 2004) or more recent (Sherriff et al., 2015) work has incorporated explicit assessment of tracer transformation in sediment un-mixing modelling. There remains, however, no widespread consensus as to the best additional and more detailed means of quantifying tracer conservativeness as a standard component of fingerprinting methodological decision-trees, meaning that the range test remains a standard step in data processing procedures (Collins et al., 2017). In the study reported here, however, a three-part assessment was used for tracer conservation. In addition, the sampling of deviate tracer values during the un-mixing modelling, using tracer distributions constructed on the basis of the sediment samples collected from the catchment outlet, provided an additional means of taking some account of potential tracer transformations (Collins et al., 2014). Collection of sediment samples from additional reaches along the channel network would permit inclusion of a more representative range of sediment tracer values and thereby of the potential for tracer conservation.

## 4 Conclusions

Sediment fingerprinting was successfully used to investigate the relative importance of unpaved recreational roads as a sediment source in the study area. This suggests that the approach has the potential to address a similar research question in other environmental settings, assuming good source discrimination can be achieved with the tracers selected. We therefore recommend consideration of the approach reported here by those investigators wanting to apportion sediment loss from unpaved roads and additional landscape sources elsewhere in the world. Three different composite signatures were selected using different statistical tests, but each signature suggested that the unpaved recreational roads dominate source contributions to the suspended sediment samples collected at the study catchment outlet. It remains important to assess the sensitivity of fingerprinting results to different composite signatures. A modified Bayesian mixing model was successfully used to estimate the relative source contributions, but where the requirements of a Bayesian approach are not satisfied by the tracer data, alternative frequentist models used by the sediment source fingerprinting research community could be applied. The findings support the targeting of management resources towards addressing the erosion of unpaved recreational roads. Management interventions need to be selected on the basis of experience elsewhere in tackling sediment loss from unpaved road systems in river catchments. Interventions will need to be implemented with due care and attention and well maintained to ensure sustained impact under ambient hydro-climatic conditions and the ongoing need for recreational access to mountainous areas neighbouring urban developments.
